# Outbreak of an infectious enteritis in western European hedgehogs (*Erinaceus europaeus*) in a rescue centre

**DOI:** 10.1007/s11259-026-11127-1

**Published:** 2026-02-26

**Authors:** María Cásero, Julia Serena, Sara Gomes-Gonçalves, João R. Mesquita, Fernanda Seixas, Ana Cláudia Coelho, Catarina Jota Baptista

**Affiliations:** 1 RIAS-ALDEIA - Wildlife Rehabilitation and Research Centre, Ria Formosa Natural Park, Olhão, Portugal; 2 School of Medicine and Biomedical Sciences (ICBAS), University of Porto, 4050-313 Porto, Portugal; 3 LAQV, REQUIMTE, Praça Coronel Pacheco, nº 15, 6º andar, 4050-453 Porto, Portugal; 4 Department of Veterinary Sciences, University of Trás-os-Montes and Alto Douro (UTAD), Vila Real, 5001-801 Portugal; 5 Animal and Veterinary Research Centre (CECAV), Associated Laboratory for Animal and Veterinary Sciences (AL4AnimalS), UTAD, Vila Real, 5001-801 Portugal; 6 Egas Moniz Centre for Interdisciplinary Research (CiiEM), Egas Moniz School of Health and Science, Caparica, 2829-511 Portugal; 7 Centre for the Research and Technology of Agro-Environmental and Biological Sciences (CITAB- Inov4Agro), UTAD, Vila Real, 5000-801 Portugal

**Keywords:** Enteritis, Erinaceus europaeus, hedgehog, Salmonella enterica, Zoonosis

## Abstract

The western-European hedgehog (*Erinaceus europaeus)* is a near-threatened mammal frequently admitted to wildlife rescue centres. In early 2025, an outbreak of haemorrhagic enteritis affected 28 hoglets and juvenile hedgehogs at the Ria Formosa Wildlife Recovery and Research Centre (RIAS) in Portugal, resulting in a mortality rate exceeding 35%. Clinical signs included appetite and weight loss, and diarrhoea (including bloody diarrhoea). Diagnostic testing (parasitology, virology, bacteriology and histopathology) was conducted and *Salmonella enterica* subsp. *enterica*, was isolated from internal organs (liver, spleen, intestine, and lung) of four necropsied individuals. Following antimicrobial susceptibility testing, treatment with trimethoprim-sulfamethoxazole associated with critical care led to clinical improvement in surviving animals. This case underscores the challenges in diagnosing and managing infectious disease outbreaks in wildlife rescue centres and highlights the zoonotic risk of diseases that can affect hedgehogs, as salmonellosis. The findings emphasise the need for enhanced surveillance, rapid diagnostics, and effective biosecurity protocols in conservation centres to ensure animal welfare, good recovery rates and mitigate public health risks.

**Supplementary Information:**

The online version contains supplementary material available at 10.1007/s11259-026-11127-1.

## Background

The western-European hedgehog (*Erinaceus europaeus*) is a small mammalian insectivore, distributed in western and central Europe, which conservation status is classified as “Near threatened”. Hedgehogs are one of the most common species found in rescue centres and seen by people in different rural and urban environments, such as private and public gardens, agricultural fields and protected areas. The reasons for admission of hedgehogs in rescue centres are diverse but anthropogenic causes play a central role in most cases, specifically due to car collision, trauma due to lawnmowers, predation by dogs, accidental poisoning, separation of orphans, and infectious diseases (Garcês et al. 2020; Gazzard et al. 2025; Jota Baptista et al. 2021; Kadlecova et al. 2023; Rasmussen et al. 2020).

Gastrointestinal signs are common in hedgehogs, especially hoglets and juveniles, and can negatively affect the rehabilitation of hedgehogs, compromising their appetite, nutritional status, weight gain, and, consequently, their recovery and wildlife reintroduction. According to the literature, several infectious agents can be associated with gastrointestinal disease in hedgehogs. These include parvovirus, particularly hedgehog chaphamaparvovirus (HhChPV) (Lanave et al. 2023), *Salmonella* sp. (Carrera et al. 2023; Lawson et al. 2018; Metiner et al. 2023), *Campylobacter jejuni*, *Escherichia coli*, *Capillaria erinacei*,* Capillaria ovoreticulata Giardia* spp. *Isospora* spp. *Brachylaemus erinacei* and *Cryptosporidium* spp. (Jota Baptista et al. 2023a, b; Krawczyk et al. 2015). Regarding the specific clinical presentation, *C. erinacei* and *C. ovoreticulata* infections are associated with mucoid and green diarrhoea. *Isospora rastegaiev* and *Isospora erinacei* are more commonly associated with haemorrhagic faeces and diarrhoea. *Brachylaemus erinacei* is related to haemorrhagic enteritis with mucoid feces or melena. Salmonellosis is a frequent disease in hoglets and juveniles, usually presenting as a sudden outbreak of diarrhoea. Post-mortem exams commonly reveal a mucohemorrhagic enteritis with congestion of the intestinal mucosa, sometimes accompanied by focal hepatomegaly, hepatic necrosis, and septicaemia (Jota Baptista et al. 2023a, b).

Most of these agents are recognised as potential causes of enteritis in humans and domestic species, revealing a zoonotic potential, even though in most cases the primary transmission route is believed to be food-borne (Jota Baptista et al. 2023a, b; Krawczyk et al. 2015; Nauerby et al. 2000; Petersen et al. 2001). Indirect contact with an animal reservoir is also described as an alternative source of infection. For instance, a study on Denmark reported that strains of *Salmonella enteritidis*, collected from hedgehogs, belong to the same clonal lineage as those isolated from infected humans (Nauerby et al. 2000). On the other hand, the zoonotic potential of some parasites has not been fully described. Many studies have suggested a limited zoonotic potential for some species of *Giardia*, since strains isolated from humans were rarely found in animals (Ryan and Cacciò 2013; Sprong et al. 2009). Similarly, although zoonotic transmission of *Cryptosporidium* spp. from domestic animals to humans has been commonly described (Monis and Thompson 2003), the extent to which wildlife species, such as hedgehogs, can act as a source for *Cryptosporidium* spp. to humans remains unclear.

Thus, this report presents the actions taken during an outbreak of severe haemorrhagic enteritis in hoglets at a Portuguese rescue centre, focusing on the potential zoonotic impact and the diagnostic approaches employed, including virological, bacteriological, parasitological, and histopathological analyses.

## Case description

### Case and housing

During January 2025, a total of 28 hoglets and juvenile hedgehogs presented gastrointestinal clinical signs, including weight loss or limited weight gain, appetite loss, diarrhoea and/or bloody diarrhoea in the Ria Formosa Wildlife Recovery and Research Centre (RIAS). RIAS is located in the Ria Formosa Natural Park (Olhão, Faro) and has over fifteen years of experience in wildlife rehabilitation. RIAS receives animals from the Algarve and Baixo Alentejo regions and is part of the integrated network of Portuguese wildlife rescue centres (ALDEIA association). Its goals include conservation, the recovery of wildlife, research, and environmental education.

As a general protocol of biosecurity and quarantine, newly arrived hedgehogs stay isolated in a box until it can be confirmed they are drinking milk on their own, and then, they are moved to slightly larger boxes with healthy hedgehogs with similar weight. Due to practical reasons related to available space, hedgehogs showing clinical signs are not usually isolated. However, if there is a suspicion of a contagious cause (e.g. infectious disease), treatment is applied to all animals in the same cage.

As feeding protocol, Royal Canin^®^ kitten milk is provided until the animals reach 100 g. From this point, milk remains available, but wet food is also introduced. At 200 g, milk feeding is discontinued, and in some cases, dry food is also introduced (in addition to the wet food). Once animals reach 300 g, fruit and chicken were added to the diet (alongside dry and wet food).

The clinical signs began to appear in animals from different cages almost simultaneously. In most cases, the problems started in the internal intensive care cages, where the animals usually spend the first days/weeks until they reach 300 g, when they are usually transferred to larger outdoor enclosures. The vast majority of hedgehogs started to have problems in the indoor boxes, but some developed problems later when they were already in the outdoor facilities. Until the middle of February, ten of these 28 hedgehogs died from gastrointestinal disease (two of them euthanised), revealing a mortality rate of more than 35% attributed to this cause.

### Sample collection

On 15th January, fecal samples from nine hedgehogs with gastrointestinal disease (V2136/24; V2160/24; V2137/24; V2190/24; V2193/24; V2195/24; V2163/24; V2138/24; and V2178/24) were collected to frozen Eppendorf tubes and sterile swabs with medium and immediately sent to virology and bacteriology analysis, respectively. Simultaneously, parasitological analysis was performed on fresh-collected feces after being sent to the external lab (INDEXX^®^ Reference Laboratory).

On 9th February, one of the hedgehogs was necropsied at RIAS (V2193/24); while three frozen (-16 °C) carcasses (V0017/25, V2195/24, and V0060/25) were sent for a complete pathological examination to the University of Trás-os-Montes e Alto Douro Histology and Surgical Pathology Lab. Samples of liver, spleen, intestine, and lung, from all four animals were collected for histopathology, and swabs in aerobic medium, were sent to bacteriology examination.

### Virology

For virology, DNA was extracted from hedgehog stool samples by diluting the fecal material (10% w/v) in phosphate-buffered saline (PBS, pH 7.2). The suspensions were centrifuged at 8,000 × g for 5 min, and 140 µl of the resulting supernatant was used for DNA extraction and purification. Extraction was performed using the QIAamp DNA Mini Kit (Qiagen, Hilden, Germany) according to the manufacturer’s instructions and automated on the QIAcube^®^ platform (Qiagen). Purified DNA was eluted in RNase-free water and stored at − 80 °C.

Molecular detection of hedgehog chaphamaparvovirus (HhChPV) was carried out using the primer set 2421_HhChPV_F and 2410_HhChPV_R, as previously described (Di Profio et al. 2024). PCR was performed on a T100 thermal cycler (Bio-Rad, Hercules, CA, USA). Reaction mixtures were prepared with the Speedy Supreme NZYTaq 2× Green Master Mix (NZYTech, Lisbon, Portugal) following the manufacturer’s protocol. The thermal cycling conditions consisted of an initial denaturation at 95 °C for 5 min, followed by 40 cycles of denaturation at 94 °C for 2 s, annealing at 60 °C for 5 s, and extension at 72 °C for 5 s, with a final extension at 72 °C for 10 min. Synthetic template oligonucleotide was designed to contain the binding sites for assay primers and used as template for positive control. PCR products were analyzed by electrophoresis on 1.0% agarose gels stained with Xpert Green Safe DNA gel dye (GRiSP^®^, Porto, Portugal). Electrophoresis was conducted at 120 V for 30 min, and DNA bands were visualized under UV light.

PCR analyses of the samples were negative, with no amplification products detected by agarose gel electrophoresis.

### Parasitology

Stool samples were collected and sent to IDEXX^®^ laboratories, where flotation, sedimentation and Baermann techniques were performed according to commonly used methodologies (Garcia 2016). For the flotation, fecal material is mixed with a solution of higher specific gravity (sugar), which leads to the migration to the surface, and the collection from the top layer for microscopic examination. For sedimentation, the supernatant is discarded, and the sediment is examined microscopically. For Baermann technique, the sample is placed on gauze in warm water; larvae actively migrate out of the material and move downward, eventually collecting at the bottom of the tube. Results were negative for all samples and the three techniques.

### Bacteriology

All collected samples were initially inoculated into non-selective pre-enrichment medium, Buffered Peptone Water (BPW), and incubated at 37 °C for 24 h. Subsequently, 1 mL of the pre-enriched culture was transferred to 10 mL of Rappaport Vassiliadis (RV) selective enrichment broth and incubating at 41.5 °C for 24 h. Following selective enrichment, a loopful of each culture was streaked onto different selective and differential agar media, including MacConkey agar, Xylose Lysine Deoxycholate (XLD) agar (Sigma-Aldrich), and CHROMagar™ Salmonella (Sigma-Aldrich). The use of non-selective pre-enrichment and differential media allowed the detection of other Enterobacterales and common enteric bacteria, thereby reducing the likelihood of overlooking alternative bacterial pathogens.

Pure isolates were subjected to a battery of phenotypic identification tests, including oxidase, catalase, sugar fermentation (glucose, lactose, maltose, sucrose, xylose, and mannitol), indole, methyl red, Voges-Proskauer, citrate utilization, Triple Sugar Iron (TSI), urease, nitrate reduction, lysine and ornithine decarboxylase, malonate utilization, and gelatinase. No other bacterial pathogens commonly associated with haemorrhagic enteritis were identified. For additional confirmation, biochemical identification was performed using the API 20E system (bioMérieux™, France), following the manufacturer’s instructions. All isolates yielded the numerical profile 6,704,552, which corresponds to *Salmonella enterica* subsp. *enterica* according to the API 20E database. The API 20E system allows reliable identification at genus and subspecies level but does not permit serovar determination; therefore, serotyping was beyond the scope of this study.

The fecal samples collected initially (V2136/24; V2160/24; V2137/24; V2190/24; V2163/24; V2138/24; and V2178/24) were negative for relevant bacteria. However, all the internal tissues samples from the dead hedgehogs collected afterwards during the necropsies (V2193/25, V2195/24, V0017/25, V0060/25) were positive for *Salmonella enterica* subsp. *enterica* through cultural, morphological, and biochemical identification methods.

The antibiogram was performed afterwards and showed sensitivity to ampicillin, amoxicillin-clavulanic acid, cefazolin, cefotaxime, ceftazidime, enrofloxacin, marbofloxacin, trimethoprim-sulfamethoxazole, tetracycline, doxycycline, amikacin, and meropenem.

### Histopathology

Tissues collected during the necropsies were processed for light microscopy using routine histological technique. Slides were stained with haematoxylin and eosin, and specific stains (Giemsa, Gram, and Ziehl-Neelsen stains) were used when necessary. Histopathology slides were observed using an optical microscope (Nikon E600^®^, Nikon Instruments Inc. Melville, NY, USA).

All the sampled animals consistently presented mesenteric lymphadenomegaly, with a marked follicular hyperplasia of the lymph nodes (V2193/24, V0017/25, V2195/24, and V0060/25). V2193/24 also presented enteritis, necrotizing hepatitis, and granulomatous lymphadenitis, negative to Ziehl-Neelsen stain. V0017/25 and V0060/25 presented verminous bronchitis. Table 1 shows the summary of the pathology and histopathology results.

**Table 1:** Macroscopic and microscopic changes found in the *Salmonella enterica* subsp. *enterica* positive animals

Internal number	Macroscopic Exam	Microscopic Exam	Main diagnosis
V2193/24	Mesenteric lymphadenomegaly	Liver lipidosis, and extramedullary haematopoiesis. Multifocal necrotizing hepatitis and thrombosis. Enteritis. Granulomatous lymphadenitis, negative for Ziehl-Neelsen staining.	Granulomatous lymphadenitis. Acute hepatitis.
V0017/25	Congestion and haemorrhage of thymus and lungs; Mesenteric lymphadenomegaly.	Parasitic bronchitis (nematodes). Extramedullary haematopoiesis in the liver and spleen	Parasitic bronchitis
V0060/25	Mesenteric lymphadenomegaly	Parasitic bronchitis (nematodes) Spleen extramedullary haematopoiesis	Parasitic bronchitis
V2195/24	Mesenteric lymphadenomegaly	Nephrosis.	Nonspecific findings

### Medical management and treatment

Due to the initial suspicion of coccidia infection, metronidazole (40 mg/kg, SC, every 24 h, for a minimum of 5 days) was administered to animals showing gastrointestinal clinical signs and/or weight loss or to those housed in a box with other animals displaying similar symptoms, prior to obtaining diagnostic results, until significant reduction of the clinical signs. Toltrazuril (10 mg/kg, PO, administered in two consecutive days in three consecutive weeks) was only introduced after metronidazole treatment failed to produce clinical improvement, and not simultaneously. By that point, affected individuals were already in poor clinical condition.

Based on the positive results of a *Salmonella enterica* subsp. *enterica* infection, Trimethoprim + Sulfamethoxazole (30 mg/kg, SC, every 12 h, for a minimum of 5 days) was administered to affected individuals, based on suspicion, until significant reduction of the clinical signs. Clinical improvement was observed once it was introduced, but unfortunately, some critically ill individuals died before treatment could be adjusted. The detailed recorded of each animal is shown in Supplementary file 1.

## Discussion and conclusion

The confirmed presence of *Salmonella enterica* subsp. *enterica*. in the affected hedgehogs at RIAS provides a clear explanation for the outbreak of haemorrhagic enteritis observed at the beginning of 2025. These results align with previous reports identifying *Salmonella enterica*, as a significant cause of enteritis in western European hedgehogs, especially in juveniles and hoglets. Clinical signs such as diarrhoea, weight loss, and sudden death are consistent with salmonellosis, which can range from mild gastroenteritis to severe systemic illness with septicaemia (Doss and Carpenter 2025). Similar outbreaks have been documented in wildlife rescue centres across Europe, including in Finland and the UK, where *Salmonella enterica* was frequently isolated from both symptomatic and asymptomatic hedgehogs (Lawson et al. 2018). Although toltrazuril was introduced under the suspicion of coccidiosis, no clinical improvement was noted during its administration, and coprological tests were negative. Recovery was observed after initiating antibiotic therapy targeting *Salmonella enterica*, which aligns with microbiological confirmation of infection. Therefore, the improvement is most plausibly attributed to antimicrobial treatment rather than toltrazuril.

Although asymptomatic salmonellosis has been described in hedgehogs, the consistent isolation of *Salmonella enterica* subsp. *enterica* from multiple internal organs (liver, spleen, intestine and lung) supports its role as the causative agent of this outbreak. Several factors likely predisposed the hedgehogs to the outbreak and may explain the progression observed (Gaffuri 2012; Lawson et al. 2018). Age‑related susceptibility is a key component: hoglets and young juveniles have immature immune systems, making them more vulnerable to enteric pathogens and less capable of containing early infection. Isolation from normally sterile tissues, in combination with histological and clinical findings, indicates systemic infection and active disease (Gaffuri 2012; Lawson et al. 2018). Moreover, it is essential to examine the management and environmental factors within the rehabilitation centre. The near-simultaneous onset of clinical signs in animals housed in different cages strongly suggests cross‑contamination. Hedgehogs admitted from diverse origins also introduce heterogeneous microbial backgrounds. Potential fomite transmission must also be considered, particularly given the intensive handling required for hoglets and juveniles. Shared equipment (including cleaning materials, weighing scales, and others) may act as mechanical vectors if not disinfected between animals. Keepers and veterinarians circulating between cages in a relatively confined space can similarly contribute to microbial spread, even if following the necessary biosecurity measures or using proper protection equipment. This is compounded by the centre’s high workflow, in which multiple animals are fed and cleaned in succession, increasing the risk of unnoticed environmental contamination (Gaffuri 2012; Đuričić and Lukač 2025). In many wildlife centres, practical limitations, such as restricted staff or lack of stricter isolation rooms, can compromise biosecurity practices. Feeding and hygiene practices likely also contributed to sustaining the outbreak (Gaffuri 2012; Đuričić and Lukač 2025). Milk replacers and wet foods provide nutrient-rich substrates that can support bacterial proliferation if storage or preparation is not carefully controlled. In this case, animals were frequently housed in groups based on weight, which is common in wildlife rehabilitation of hedgehogs, but group feeding inherently increases fecal–oral exposure between individuals. Furthermore, symptomatic hedgehogs were not routinely isolated due to lack of space, facilitating possible pathogen circulation (Gaffuri 2012; Đuričić and Lukač 2025).

Negative results in initial faecal samples and subsequent detection in carcasses can occur with this infectious agent in wildlife species. Intermittent shedding is commonly described in small mammals, particularly in juveniles, where bacterial excretion may fluctuate with disease stage and systemic spread (Goethem 2026). For instance, sampling timing can represent a limitation, i.e. the first samples were collected shortly after the onset of clinical signs, whereas the bacteria were detected when animals reached advanced disease, at necropsy, when bacterial loads in tissues were substantially higher. In addition, stress and rapid deterioration can modify gastrointestinal motility and local bacterial burdens, further reducing detection in faeces while allowing the pathogen to disseminate systemically, which is consistent with the *Salmonella*-positive organ cultures obtained during necropsy. Technical constraints may also have contributed, as faecal samples from live animals are more prone to contamination, degradation, or insufficient volume compared with organ samples collected post‑mortem under controlled laboratory conditions.

These findings also reinforce the importance of routine virology, parasitology and bacteriology screening in wildlife rehabilitation settings, particularly during episodes of gastrointestinal disease, and highlight the need for rapid diagnostic and containment measures to prevent new infections and improve survival outcomes. This case also highlights the diagnostic and therapeutic challenges faced by wildlife rehabilitation centres (Burroughes et al. 2021; Gazzard et al. 2025). The lack of specific clinical signs, the rapid progression of disease, and the limited availability of species-specific diagnostic tools often hinder timely and effective intervention. Moreover, the absence of macroscopic lesions in most necropsied animals, aside from haemorrhagic enteritis, emphasises the need for comprehensive histopathological and microbiological analyses to identify causative agents (Doss and Carpenter 2025).

The zoonotic potential of pathogens carried by hedgehogs adds another layer of complexity. While the primary transmission route for agents like *Salmonella* sp. and *Campylobacter* spp. is foodborne, indirect contact with infected wildlife or contaminated environments is a recognised risk (Ruszkowski et al. 2021). The detection of clonal lineages of *Salmonella enterica* subsp. *enterica* in both hedgehogs and humans in Denmark exemplifies this risk (Nauerby et al. 2000).

In conclusion, the outbreak at RIAS serves as a stark reminder of the importance of integrated conservation and health strategies. The importance of reinforcing early isolation of symptomatic individuals, stricter disinfection protocols for equipment and surfaces, and revising management and feeding protocols are crucial to minimise contamination events. While resource constraints represent a limitation in the majority of rescue centres, targeted improvements in these areas may substantially reduce the likelihood of future outbreaks. Finally, enhanced disease surveillance, public education on zoonotic risks, and investment in wildlife health infrastructure are critical to safeguarding hedgehog health, welfare and conservation, as well as public health, since hedgehogs commonly inhabit urban and peri-urban environments. Strengthening diagnostic capacity and establishing standardised protocols for outbreak investigation in wildlife centres are essential steps toward improving outcomes.

## Supplementary Information

Below is the link to the electronic supplementary material.

Supplementary Material 1 (DOCX 32.3 KB)

## Data Availability

The datasets generated during the current study are available from the corresponding author on reasonable request.
