# Supplementary material for: Outbreak of an infectious enteritis in western European hedgehogs (Erinaceus europaeus) in a rescue centre
Source: Vet Res Commun. 2026 Feb 26;50(3):176. doi: 10.1007/s11259-026-11127-1 (PMC12945935; doi:10.1007/s11259-026-11127-1)
Supplement: Supplementary file 1 — Supplementary Material 1 (DOCX 32.3 KB) [file 11259_2026_11127_MOESM1_ESM.docx]

Supplementary file 1 - Outbreak of an infectious enteritis in western European hedgehogs (*Erinaceus europaeus*) in a rescue centre.

Table 1 – Clinical record of rescued hedgehogs followed during the outbreask. Dates are presented as dd/mm/yy. (C- coprology; P- pathology; B- bacteriology; V – virology)

| ID | Admission | Starting weight | Key clinical signs | Diagnostics | Treatments | Outcome |
| --- | --- | --- | --- | --- | --- | --- |
| V1870/24 | 04/10/24 | 68 g | Bloody diarrhea (11/01/25) | _ | Metronidazole | Released |
| V1925/24 | 14/10/24 | 112 g | Blood in feces (09/12/24) | _ | Metronidazol Cotrimoxazol | Euthanised 01/04/25 |
| V2136/24 | 07/12/24 | 106 g | _ | C: negative  B: negative  V: negative | Metronidazol | Released |
| V2137/24 | 07/12/24 | 93 g | Bloody diarrhea mid‑Jan | C: negative  B: negative  V: negative | Metronidazol | Released |
| V2138/24 | 07/12/24 | 150 g | Diarrhea + blood in feces (30/12/24) | C: negative  B: negative  V: negative | Metronidazol | Died 01/01/25 |
| V2160/24 | 17/12/24 | 136 g | _ | C: negative  B: negative  V: negative | Metronidazol | Released |
| V2178/24 | 23/12/24 | 344 g | Blood in feces (15/01/25) | C: negative  B: negative  V: negative | Metronidazol | Died |
| V2190/24 | 27/12/24 | 76 g | Blood in feces (24/01/25) | C: negative  B: negative  V: negative | Metronidazol Toltrazuril | Euthanised 10/02/25 |
| V2193/24 | 27/12/24 | 70 g | Diarrhea (24/01/25) | C: negative  B: *Salmonella* sp.  V: negative  P: Mesenteric lymphadenomegaly. Granulomatous lymphadenitis. Acute hepatitis. Enteritis. | Metronidazol Toltrazuril | Died 06/02/25 |
| V2195/24 | 28/12/24 | 96 g | Bloody diarrhea (24/01/25) | C: negative  B: *Salmonella* sp.  V: negative  P: Mesenteric lymphadenomegaly. | Metronidazol Toltrazuril | Died 02/02/25 |
| V0008/25 | 02/01/25 | 120 g | _ | _ | Metronidazol | Released |
| V0009/25 | 02/01/25 | 104 g | Weight loss | _ | Metronidazol | Died 31/01/25 |
| V0011/25 | 03/01/25 | 190 g | _ | C: negative | Amoxiclav Metronidazol | Released |
| V0014/25 | 03/01/25 | 104 g | _ | _ | Metronidazol | Released |
| V0017/25 | 06/01/25 | 140 g | Blood in feces (24/01/25) | B: *Salmonella* sp.  P: Mesenteric lymphadenomegaly. Congestion and haemorrhage of thymus and lungs. Parasitic bronchitis. | Metronidazol Toltrazuril | Died 02/02/25 |
| V0049/25 | 12/01/25 | 130 g | Bloody diarrhea (24/01/25) | _ | Metronidazol | Died 28/01/25 |
| V0057/25 | 14/01/25 | 92 g | _ | _ | Metronidazol  Cotrimoxazol | Released |
| V0058/25 | 15/01/25 | 92 g | Weight loss | _ | Fluids | Died 27/01/25 |
| V0060/25 | 16/01/25 | 180 g | Blood in feces (24/01/25) | B: *Salmonella* sp.  P: Mesenteric lymphadenomegaly. Parasitic bronchitis | Metronidazol | Died 01/02/25 |
| V0102/25 | 24/01/25 | 122 g | Blood in feces (10/02/25) | _ | Metronidazol Cotrimoxazol | Released |
| V0116/25 | 27/01/25 | 92 g | _ | _ | Cotrimoxazol | Released |
